# Effects of empagliflozin versus placebo on cardiac sympathetic activity in acute myocardial infarction patients with type 2 diabetes mellitus: the EMBODY trial

**DOI:** 10.1186/s12933-020-01127-z

**Published:** 2020-09-25

**Authors:** Wataru Shimizu, Yoshiaki Kubota, Yu Hoshika, Kosuke Mozawa, Shuhei Tara, Yukichi Tokita, Kenji Yodogawa, Yu-ki Iwasaki, Takeshi Yamamoto, Hitoshi Takano, Yayoi Tsukada, Kuniya Asai, Masaaki Miyamoto, Yasushi Miyauchi, Eitaro Kodani, Masahiro Ishikawa, Mitsunori Maruyama, Michio Ogano, Jun Tanabe, EMBODY trial investigators, Reiko Shiomura, Isamu Fukuizumi, Junya Matsuda, Satsuki Noma, Hideto Sangen, Hidenori Komiyama, Yoichi Imori, Shunichi Nakamura, Jun Nakata, Hideki Miyachi, Gen Takagi, Takahiro Todoroki, Takeshi Ikeda, Tomoyo Miyakuni, Ayaka Shima, Masato Matsushita, Hirotake Okazaki, Akihiro Shirakabe, Nobuaki Kobayashi, Masamitsu Takano, Yoshihiko Seino, Yugo Nishi, Keishi Suzuki, Junsuke Shibuya, Tsunenori Saito, Hiroyuki Nakano, Morisawa Taichirou, Erito Furuse, Kenji Nakama, Yusuke Hosokawa, Ippei Tsuboi, Hidekazu Kawanaka

**Affiliations:** 1 Department of Cardiovascular Medicine, Nippon Medical School, 1-1-5, Sendagi, Bunkyo-ku, Tokyo, 113-0022 Japan; 2 Department of Cardiovascular Medicine, Nippon Medical School Chiba Hokuso Hospital, Chiba, Japan; 3 Department of Cardiovascular Medicine, Nippon Medical School Tama Nagayama Hospital, Tokyo, Japan; 4 Department of Cardiovascular Medicine, Nippon MEDICAL School Musashi Kosugi Hospital, Tokyo, Japan; 5 Department of Cardiovascular Medicine, Shizuoka Medical Center, Shizuoka, Japan

**Keywords:** Acute Myocardial Infarction, Heart rate variability, Heart rate turbulence, Sodium–glucose cotransporter 2 inhibitor, Sudden cardiac death, Randomized Controlled Trial

## Abstract

**Background:**

Protection from lethal ventricular arrhythmias leading to sudden cardiac death (SCD) is a crucial challenge after acute myocardial infarction (AMI). Cardiac sympathetic and parasympathetic activity can be noninvasively assessed using heart rate variability (HRV) and heart rate turbulence (HRT). The EMBODY trial was designed to determine whether the Sodium–glucose cotransporter 2 (SGLT2) inhibitor improves cardiac nerve activity.

**Methods:**

This prospective, multicenter, randomized, double-blind, placebo-controlled trial included patients with AMI and type 2 diabetes mellitus (T2DM) in Japan; 105 patients were randomized (1:1) to receive once-daily 10-mg empagliflozin or placebo. The primary endpoints were changes in HRV, e.g., the standard deviation of all 5-min mean normal RR intervals (SDANN) and the low-frequency–to–high-frequency (LF/HF) ratio from baseline to 24 weeks. Secondary endpoints were changes in other sudden cardiac death (SCD) surrogate markers such as HRT.

**Results:**

Overall, 96 patients were included (46, empagliflozin group; 50, placebo group). The changes in SDANN were + 11.6 and + 9.1 ms in the empagliflozin (P = 0.02) and placebo groups (P = 0.06), respectively. Change in LF/HF ratio was – 0.57 and – 0.17 in the empagliflozin (P = 0.01) and placebo groups (P = 0.43), respectively. Significant improvement was noted in HRT only in the empagliflozin group (P = 0.01). Whereas intergroup comparison on HRV and HRT showed no significant difference between the empagliflozin and placebo groups. Compared with the placebo group, the empagliflozin group showed significant decreases in body weight, systolic blood pressure, and uric acid. In the empagliflozin group, no adverse events were observed.

**Conclusions:**

This is the first randomized clinical data to evaluate the effect of empagliflozin on cardiac sympathetic and parasympathetic activity in patients with T2DM and AMI. Early SGLT2 inhibitor administration in AMI patients with T2DM might be effective in improving cardiac nerve activity without any adverse events.

*Trial Registration:* The EMBODY trial was registered by the UMIN in November 2017 (ID: 000030158). UMIN000030158; https://upload.umin.ac.jp/cgi-open-bin/ctr_e/ctr_view.cgi?recptno=R000034442.

## Background

In recent large randomized placebo-controlled trials, sodium–glucose cotransporter 2 (SGLT2) inhibitors have been found to decrease cardiovascular (CV) events, particularly as secondary prevention [1–3]. Furthermore, in all the trials, hospitalizations for heart failure were reduced from the early stage of the trials [1–3]. In the EMPAREG OUTCOME trial, cardiovascular deaths, including sudden cardiac death (SCD), were reduced from the early stage in the empagliflozin group, and fatal arrhythmia was inhibited in high-risk patients [1]. This is difficult to explain based on the inhibition of arteriosclerosis and long-term pathological improvements via the blood glucose-lowering effect of SGLT2 inhibitors. Furthermore, SGLT2 inhibitors have been found to have multiple effects in addition to the blood glucose-lowering effect; however, these have not yet been fully elucidated [4]. More specifically, it is unclear how SGLT2 inhibitors affect cardiac sympathetic or parasympathetic nerve activities. Protection from lethal ventricular arrhythmias leading to SCD is one of the most important challenges after acute myocardial infarction (AMI) [5]. Both increased cardiac sympathetic activity and decreased cardiac parasympathetic activity were associated with poor prognosis and fatal arrhythmias [6]. To date, noninvasive techniques such as the T-wave alternans (TWA), late potentials (LP), heart rate turbulence (HRT), and heart rate variability (HRV) have been developed for evaluating both cardiac sympathetic and parasympathetic activities. It was crucial to evaluate the effects of empagliflozin on the improvement in cardiac nerve activity in the early phase of AMI. Therefore, the EMBODY trial was conducted to elucidate the mechanisms by which empagliflozin reduces CV-related deaths, including SCD in patients with AMI and type 2 diabetes mellitus (T2DM) [7]. The aim of the EMBODY trial was to determine how the SGLT2 inhibitor affects cardiac sympathetic and parasympathetic activities in patients with AMI and T2DM.

## Methods

### Trial design

The EMBODY trial was a prospective, multicenter, randomized, double-blind, placebo-controlled trial in patients with AMI and T2DM in Japan. The detailed methods have been published previously [7]. A total of 105 patients were randomized (1:1) to receive once-daily empagliflozin (10 mg) or once-daily placebo. We aimed to assess the beneficial effects of empagliflozin on cardiac nerve activity in comparison with a placebo in relation to lethal ventricular tachyarrhythmias measured by Holter electrocardiography (ECG) (SCM-8000 Fukuda Denshi Co., Ltd. Tokyo, Japan) and 123I-*meta*-iodobenzylguanide (MIBG) scintigraphy.

### Trial population and follow-up

Recruitment for the trial began in February 2018 and ended in March 2019. The inclusion and exclusion criteria had been published [7]. Patients with AMI and T2DM were randomly assigned into an empagliflozin (10 mg/day) or a placebo group, both add-ons to conventional therapy 2 weeks after the onset of AMI based on allocation factors, baseline HbA1c value (˂7.0% or ≥ 7.0%) and max creatine kinase (CK) (˂3000 IU/L or ≥ 3000 IU/L) by a dynamic allocation method. Post-randomized follow-up visits were scheduled at 4, 12, and 24 weeks. Patients also received post-AMI treatment with β-blockers, antiplatelet therapy, statins, and renin–angiotensin system inhibitors in accordance with local guidelines [8, 9]. Throughout the trial period, investigators were encouraged to treat other cardiovascular risk factors, including dyslipidemia and hypertension, to provide the best available standard of care. Data from 24-h continuous ambulatory digital Holter ECG recordings were measured for specific ECG markers, such as HRV, HRT, LP, and TWA, using the systems manufactured by Fukuda Denshi Co., Ltd. (Tokyo, Japan). All patients underwent Holter ECG monitoring with daily activities under a stable state at baseline after 24 weeks.

### Trial endpoints

The primary endpoint of this trial was the change in HRV from baseline to 24 weeks. Secondary endpoints were the changes from baseline to 24 weeks in the following measurements in the empagliflozin group compared with those in the placebo group throughout the trial period.

1. TWA, LP, and HRT assessed using ambulatory ECG (SCM-8000)
2. Cardiac sympathetic activity assessed using 123I-MIBG scintigraphy

In addition, we compared the changes from baseline to 24 weeks in other variables, including glycemic and lipid profiles, uric acid, estimated glomerular filtration rate (eGFR), body weight (BW), blood pressure (BP), and safety parameters, such as adverse events (cardiovascular death, nonfatal MI, nonfatal stroke, or hospitalization for heart failure) after 24 weeks treatment.

### Analyses of the cardiac sympathetic activity

#### Measurement of HRV

The HRV provided important information about the sympathovagal balance (low-frequency power; LF, high-frequency power; HF) of the heart. Traditionally, HRV was analyzed using time and frequency domain methods [7].

Time domain analysis included the following:

1. Mean RR interval for 24 h (mean NN)
2. Standard deviation of normal RR intervals (SDNN)
3. Standard deviation of all 5-min mean normal RR intervals (SDANN)
4. Square root of the mean of the sum of the squares of differences between adjacent RR intervals (r-MSSD)
5. Percentage of adjacent RR intervals differing by > 50 ms (pNN50)

Frequency domain analysis included the following:

1. HF (0.15–0.4 Hz)
2. LF (0.04–0.15 Hz)
3. Sympathovagal balance (LF/HF ratio)

### Measurement of HRT

HRT parameters included turbulence onset (TO) and turbulence slope (TS), which were determined by a previous study [10]. TO ≥ 0% and TS ≤ 2.5 ms/RR intervals are considered abnormal. HRT values are usually classified into three categories: (1) HRT category 0 indicates that TO and TS are normal; (2) HRT category 1 indicates that either TO or TS is abnormal; and (3) HRT category 2 indicates that both TO and TS are abnormal. In the present study, we analyzed the improvement in HRT over 24 weeks in the two groups.

#### Measurement of LP

LP were analyzed for 24 h using a developed signal-averaging system (SCM-8000, Fukuda Denshi Co., Ltd.) that can automatically analyze LP every 30 min using data from a digital Holter ECG recorder (FM-180, Fukuda Denshi Co., Ltd.). Three parameters were assessed using a computer algorithm: filtered QRS duration (fQRS), root mean square voltage of the terminal 40 ms of the filtered QRS complex (RMS40) and duration of low-amplitude signals (< 40 μV) in the terminal, filtered QRS complex (LAS40). LP were considered positive when two of the three criteria (fQRS > 135 ms, RMS40 < 15 μV, and LAS40 > 39 ms) were met over 24 h [11, 12].

#### Measurement of TWA

TWA was simultaneously measured, and the maximum TWA magnitude (max TWA) over 24 h was determined using the spectral method, as previously described [13]. In the present study, we statistically analyzed the continuous variables.

### Statistical considerations

#### Sample size estimation

Sample size was calculated for LF/HF ratio, one of the primary endpoints. Owing to a lack of previous studies examining the effects of SGLT2 inhibitors on cardiac sympathetic or parasympathetic activity, it was estimated that the mean difference between the empagliflozin and placebo group from baseline to 24 weeks in the Ln LF/HF (ms2) is 0.3 and the SD is 0.5 (obtained from previous studies using a similar approach) [14, 15]. When the significance level is 5% (two-sided), a sample size of 88 total patients provide a power of 80% for comparison. It was estimated that at least 10% of randomized patients will not undergo treatment or will have a missing baseline value and/or a missing post-baseline value, thereby resulting in their exclusion from the analysis. Consequently, we assumed that a total of 98 patients should be enrolled in this trial.

#### Statistical analyses

All continuous values and categorical variables were expressed as mean ± standard deviation and the number and percentage of patients, respectively. The full analysis and safety analysis set included all enrolled participants who received their assigned therapy at least once after randomization. Efficacy analyses for all values were performed in the full analysis set. Between-group and intergroup comparisons of changes from baseline to 24 weeks for primary outcomes were also based on a linear regression model adjusted for allocation factors (ANCOVA) as covariants. For other outcomes, a t-test was used to analyze continuous variables, and Wilcoxon rank test was used for categorical variables. Mixed-effects model repeated measures (MMRM) analysis was used to compare the changes in BW, BP, HbA1c, eGFR, hematocrit, uric acid, glycemic, and lipid parameters, and serum ketone bodies from baseline to 24 weeks. In addition, we conducted a multiple regression analysis for the change in HRV. A two-sided probability value of *P *< 0.05 was considered statistically significant. All statistical analyses were performed using SAS version 9.4 (SAS Institute, Cary, NC, USA).

### Ethics approval and consent to participate

The EMBODY trial was registered at the UMIN in November 2017 (ID: 000030158). Ethics approval was obtained from the local institutional review board of each participating center and the trial complied with the Declaration of Helsinki. All participants provided written informed consent prior to study enrollment. The local institutional review boards and independent ethics committees approved the trial protocol. The trial was conducted in full compliance with the articles of the Declaration of Helsinki and according to the Ethical Guidelines for Medical and Health Research Involving Human Subjects established by the Ministry of Health, Labor, and Welfare and the Ministry of Education, Culture, Sports, Science, and Technology in Japan. After initial screening for eligibility using prior medical records, each patient was provided an adequate explanation of the trial plan before obtaining written informed consent from them.

## Results

**Fig. 1:**
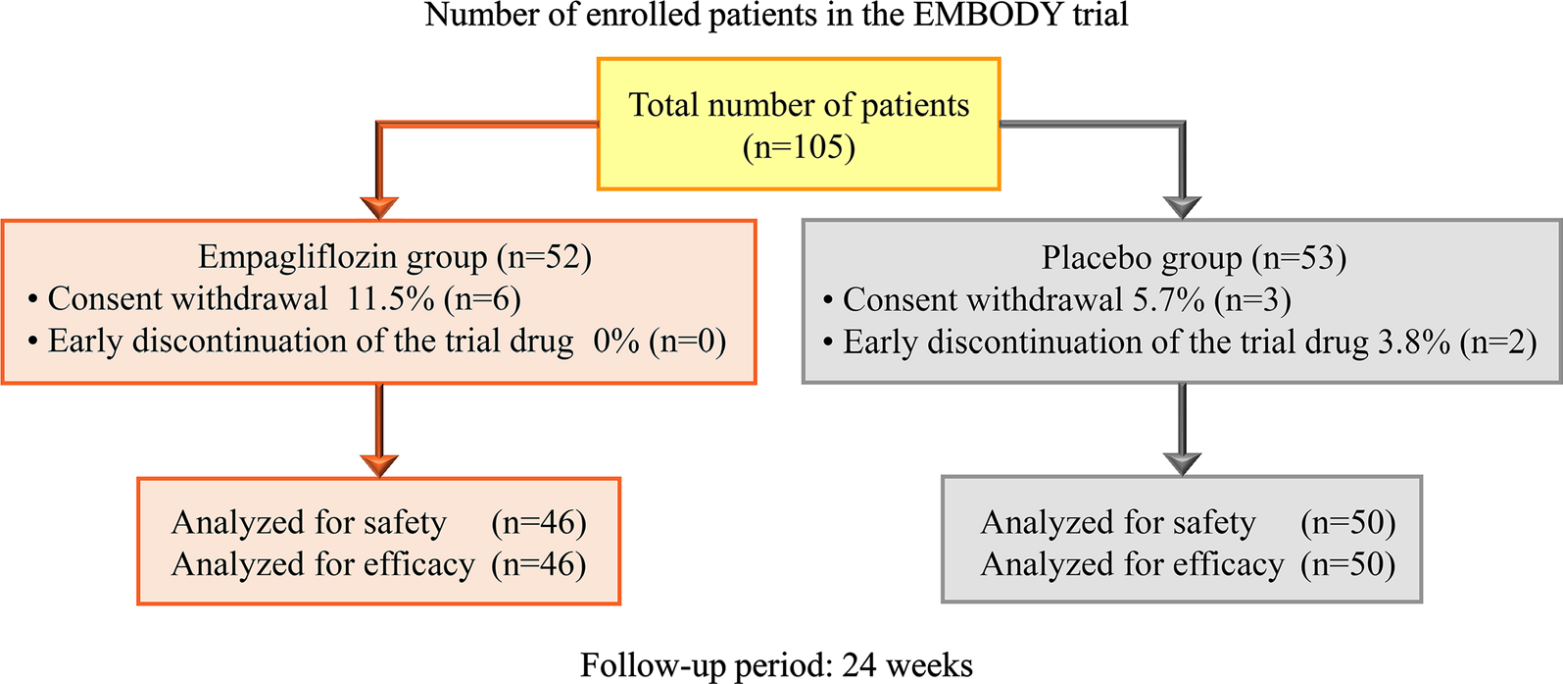
A total of 105 patients met the inclusion criteria and were randomized. Six patients in the empagliflozin group and three patients in the placebo group were excluded because of consent withdrawal before medication begun. Therefore, 96 patients were included in the full analysis set (46 in the empagliflozin group and 50 in the placebo group)

A total of 105 AMI patients with T2DM gave informed consent to participate in the trial between February 2018 and March 2019. Of whom, 105 met the inclusion criteria and were randomized (Fig. 1). Six patients in the empagliflozin group and three patients in the placebo group withdrew their consent and were thus excluded before the medication initiated. Therefore, 96 patients were finally included in the full analysis population (46 in the empagliflozin group and 50 in the placebo group). Baseline characteristics were not significantly different between the treatment groups (Table 1).

**Table 1:** Baseline demographics, characteristics, clinical and pharmacotherapy history

Variable	Empagliflozin, n = 46 [n (%)]	Placebo, n = 50 [n (%)]	P
Male, n (SD)	38 (82.6)	39 (78.0)	0.62
age, years (SD)	63.9 (10.4)	64.6 (11.6)	0.73
Body weight, kg (SD)	70.1 (13.7)	68.1 (14.4)	0.49
BMI, kg/m^2^ (SD)	25.2 (3.7)	25.2 (4.1)	0.99
DM duration, months (SD)	38.3 (43.4)	32.4 (43.3)	0.51
Current smoker, n (%)	24 (52.2)	27 (54.0)	0.92
Systolic blood pressure, mmHg (SD)	129.7 (11.9)	123.1 (15.7)	0.11
Heart rate, bpm (SD)	70.3 (11.0)	71.5 (11.4)	0.78
Medical history			
Cerebrocardiovascular disease, n (%)	7 (15.2)	11 (22.0)	0.44
Hypertension, n (%)	38 (82.6)	39 (78.0)	0.62
Dyslipidemia, n (%)	34 (73.9)	36 (72.0)	1.00
Culprit lesion			
Left anterior descending artery, n (%)	25 (54.3)	34 (68.0)	0.35
Blood sampling test			
Max CK, IU/L (SD)	2080.7 (2461.6)	2358.7(2829.1)	0.61
HbA1c, % (SD)	6.82 (1.00)	6.89 (0.92)	0.73
LDL-C, mg/dL (SD)	87.5 (29.6)	87.8 (29.1)	0.96
HDL-C, mg/dL (SD)	45.4 (12.7)	46.0 (10.2)	0.78
Triglycerides, mg/dL (SD)	161.7 (119.7)	135.9 (54.4)	0.18
Uric acid, mg/dL (SD)	5.8 (1.4)	5.7 (1.5)	0.94
Creatinine, mg/dL (SD)	0.92 (0.2)	0.92 (1.2)	0.39
eGFR, mL/min/1.73 m^2^ (SD)	64.6 (15.0)	66.1 (15.7)	0.62
Hematocrit, % (SD)	40.5 (4.6)	40.3 (4.2)	0.86
NT-pro BNP, pg/mL (SD)	1028.7 (1105.6)	1270.6 (1521.0)	0.45
Medical therapy			
β-blocker, n (%)	41 (89.1)	38 (76.0)	0.11
ARB, n (%)	22 (47.8)	19 (38.0)	0.41
ACEI, n (%)	23 (50.0)	28 (56.0)	0.68
Statin, n (%)	44 (95.7)	48 (96.0)	1.00
Spironolactone, n (%)	11 (23.9)	12 (24.0)	1.00
Diuretics, n (%)	8 (17.4)	11 (22.0)	0.62
Metformin, n (%)	7 (15.2)	6 (12.0)	0.77
DPP-4 inhibitor, n (%)	20 (43.5)	23 (46.0)	0.84
ASA/P2Y12 Inhibitor, n (%)	46 (100)	50 (100)	1.00
DOAC, n (%)	3 (6.5)	3 (6.0)	1.00

n (SD), number (standard deviation); A1c, glycated hemoglobin; ACEI, angiotensin-converting enzyme inhibitor; ARB, angiotensin-receptor blocker; ASA, acetylsalicylic acid; BMI: body mass index; CK, creatine kinase; DM, diabetes mellitus; DOAC, direct oral anticoagulant; DPP-4, Dipeptidyl Peptidase-4; eGFR, estimated glomerular filtration rate; HDL-C, high-density lipoprotein cholesterol; LDL-C, low-density lipoprotein cholesterol; NT-Pro BNP, N-terminal *pro* b-type natriuretic peptide

### Holter ECG

Table 2 presents the HRV results as the primary endpoint obtained by Holter ECG for the two groups. There were somewhat differences in HRV and HRT values at baseline between the two groups; however, they were not statistically significant. The change in SDANN was +11.6 ms (P = 0.02) in the empagliflozin group and + 9.1 ms (P = 0.06) in the placebo group, adjusted difference of 2.5 ms, 95% confidence interval (CI) − 9.5 to 14.5 ms (P = 0.68). The change in r-MSSD was + 6.5 ms (P = 0.01) in the empagliflozin group and + 2.3 ms (P = 0.35) in the placebo group, adjusted difference of 4.2 ms, 95% CI − 2.0 to 10.4 ms (P = 0.19). The change in HF was +583.1 msec^2^ (P = 0.04) in the empagliflozin group and +83.7 msec^2^ (P = 0.76) in the placebo group, adjusted difference of 499.4 msec^2^, 95% CI − 195.4 to 1194.2 msec^2^ (P = 0.16). The change in LF/HF ratio was –0.57 (P = 0.01) in the empagliflozin group and –0.17 (P = 0.43) in the placebo group, adjusted difference –0.40, 95% CI − 0.94 to 0.13 (P = 0.14). In HRV, these three indicators significantly improved only in the empagliflozin group, although the intergroup comparison revealed no significant difference.

**Table 2:** HRV parameters’ changes following empagliflozin exposure or placebo (baseline–24 weeks), assessed using Holter ECG

Parameter	Empagliflozin (n = 46)			Placebo (n = 50)			Adjusted difference Between groups (95% CI)		P
	Baseline	24 weeks	P	Baseline	24 weeks	P			
HRV									
Average NN, msec	882.4 (130.1)	889.0 (131.1)	0.43	866.7 (128.6)	884.7(99.2)	0.15	−11.6	(− 57.1, 33.9)	0.61
SDNN, msec	101.1 (37.4)	112.8 (46.1)	< 0.01	102.5 (36.9)	113.2 (33.6)	< 0.01	0.9	(− 10.1, 12.0)	0.87
SDANN, msec	81.0 (27.0)	92.7 (28.9)	0.02	82.2 (28.8)	91.4 (24.2)	0.06	2.5	(− 9.5, 14.5)	0.68
r-MSSD, msec	34.2 (54.8)	40.7 (67.6)	0.01	39.2 (54.4)	41.5 (56.0)	0.35	4.2	(− 2.0, 10.4)	0.19
pNN50, %	8.6 (18.4)	8.7 (17.7)	0.92	9.3 (18.9)	10.2 (18.8)	0.38	−0.8	(− 3.4, 1.8)	0.55
HF, msec^2^	1057.3 (4700.3)	1623.2 (6923.6)	0.04	1136.9 (4240.3)	1200.1 (4280.8)	0.76	499.4	(− 195.4, 1194.2)	0.16
LF, msec^2^	748.6 (2321.6)	1082.0 (3584.3)	0.05	818.4 (2160.3)	914.2 (2161.6)	0.60	235.6	(− 151.6, 622.7)	0.23
LF/HF ratio	2.77 (2.21)	2.37 (1.55)	0.01	2.09 (1.31)	2.09 (1.20)	0.43	−0.40	(− 0.94, 0.13)	0.14

Data are expressed as mean (SD) and analyzed using ANCOVA adjusting for allocation factors

CI, Confidence interval; HRV, heart rate variability; SDNN, Standard deviation of normal RR intervals; SDANN, Standard deviation of all 5 min mean normal RR intervals; r-MSSD, Square root of the mean of the sum of the squares of differences between adjacent RR intervals; pNN50, Percentage of adjacent RR intervals differing by > 50 ms; HF, high-frequency power; LF, low-frequency power

**Fig. 2:**
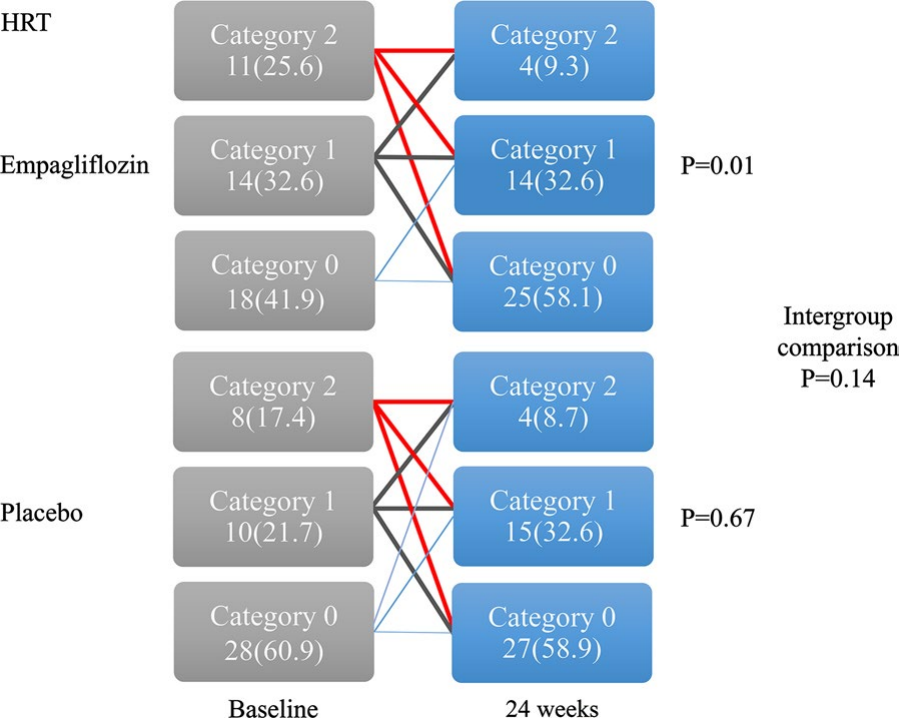
Changes from baseline in the heart rate turbulence. The category of heart rate turbulence reflecting abnormal autonomic nerve activity improved significantly in the empagliflozin group only. Intergroup comparison revealed no significant difference

**Fig. 3:**
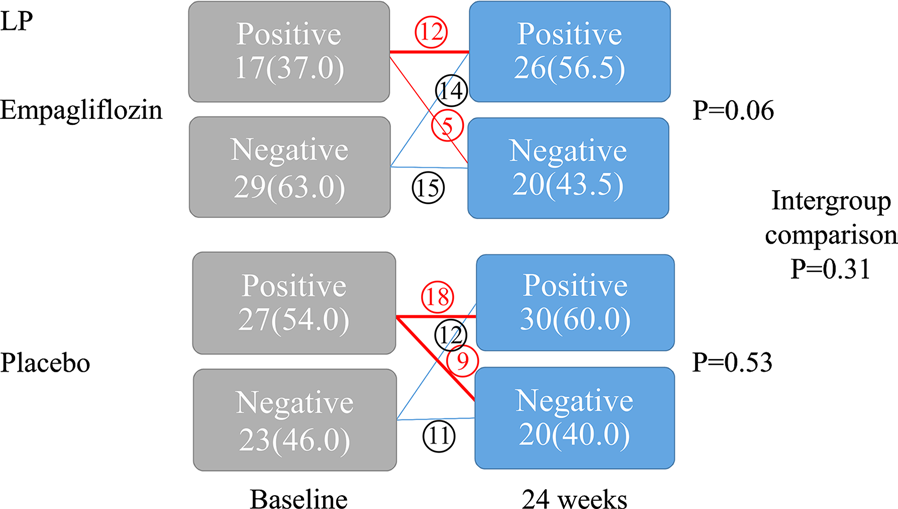
Changes from baseline in the late potentials. Intragroup and intergroup comparison revealed no significant change in late potentials as an indicator of depolarization

As another index reflecting cardiac parasympathetic activity, a significant improvement in the HRT was observed only in the empagliflozin group (P = 0.01) (Fig. 2). Intragroup and intergroup comparison revealed no significant change in LP as an indicator of depolarization (Fig. 3). Furthermore, TWA, which is an indicator of repolarization abnormality was 8.9 ± 14.0 µV at 0 weeks at baseline and 7.2 ± 10.5 µV (P = 0.45) at 24 weeks in the empagliflozin group and 8.1 ± 11.5 µV at 0 weeks at baseline and 6.3 ± 8.5 µV (P = 0.27) at 24 weeks in the placebo group, with no significant difference observed between the two groups (P = 0.97).

### Other end points

Intragroup comparison of the delayed period H/M ratio and wash-out ratio on MIBG myocardial scintigraphy improved in both the groups, and the intergroup comparison revealed no significant difference (Additional file 1).

**Fig. 4:**
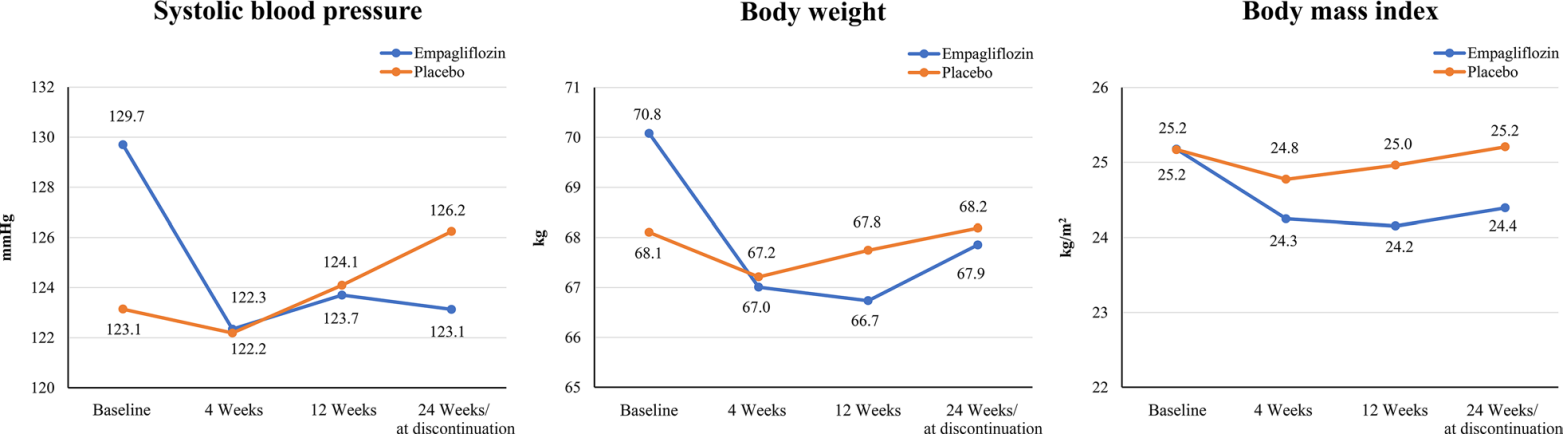
Changes from baseline in systolic blood pressure, body weight, and body mass index. The shifts in systolic blood pressure, body weight, and body mass index during the trial period are presented. Compared with the placebo group, the empagliflozin group showed an early improvement with a significant difference (Intergroup; P = 0.0005, P = 0.0002 and P = 0.0002, respectively)

Over the course of the trial period, the parameters measured during four visits were analyzed by MMRM. The BP, BW, and body mass index (BMI) were significantly decreased in the empagliflozin group compared with those in the placebo group (Fig. 4). The blood sampling test results are presented in Table 3. During the trial period, no difference was observed between the groups in terms of HbA1c levels. Compared with the placebo group, the empagliflozin group exhibited significantly higher hematocrit levels and lower uric acid levels at 24 weeks (Intergroup; P < 0.0001). Furthermore, the eGFR was significantly decreased only in the placebo group, whereas N-terminal pro b-type natriuretic peptide (NT-Pro BNP) levels improved in both groups. Ketone bodies in the blood tended to increase in the empagliflozin group compared with the placebo group (P = 0.05). Furthermore, nonHDL cholesterol and ALT levels were decreased after 24 weeks only in the empagliflozin group, whereas γ-GTP and serum creatinine levels were increased only in the placebo group (Table 3).

**Table 3:** Blood sampling test changes following empagliflozin exposure or placebo from baseline to 24 weeks

	Empagliflozin (n = 46)			Placebo (n = 50)			Intergroup
	Baseline	24 weeks	*P*	Baseline	24 weeks	*P*	*P*
HbA1c (%)	6.8 ± 1.0	6.6 ± 0.9	0.10	6.9 ± 0.9	6.8 ± 1.0	0.34	0.50
Hematocrit (%)	40.5 ± 4.6	44.2 ± 3.9	< 0.001	40.3 ± 4.2	40.5 ± 4.2	0.81	< 0.0001
Uric acid (mg/dL)	5.8 ± 1.4	4.9 ± 1.4	< 0.001	5.7 ± 1.5	5.8 ± 1.5	0.82	< 0.0001
eGFR (equivalent) (mL/min/1.73 m^2^)	64.6 ± 15.0	64.4 ± 16.8	0.84	66.1 ± 15.7	62.8 ± 15.4	0.02	0.10
Serum creatinine (mg/dL)	0.92 ± 0.19	0.94 ± 0.25	0.44	0.89 ± 0.20	0.94 ± 0.21	0.01	0.24
NT-pro BNP (pg/mL)	1028.7 ± 1105.6	370.3 ± 530.9	< 0.001	1270.6 ± 1521.0	673.7 ± 1151.1	< 0.001	0.91
Ketone bodies in the blood (venous blood) (μmol/L)	69.7 ± 51.8	119.3 ± 169.2	0.12	81.9 ± 70.9	61.2 ± 35.7	0.20	0.05
LDL-C (mg/dL)	87.5 ± 29.6	79.4 ± 21.3	0.06	87.8 ± 29.1	81.0 ± 25.0	0.09	0.83
HDL-C (mg/dL)	45.4 ± 12.7	49.8 ± 13.0	< 0.001	46.0 ± 10.2	49.2 ± 9.2	0.01	0.52
TG (mg/dL)	161.7 ± 119.7	145.5 ± 75.0	0.25	135.9 ± 54.3	141.0 ± 59.1	0.67	0.30
Non-HDL-C (mg/dL)	113.7 ± 40.5	102.3 ± 21.3	0.04	112.4 ± 32.2	105.2 ± 30.4	0.15	0.65
AST (U/L)	24.7 ± 7.9	23.2 ± 8.5	0.30	24.4 ± 10.3	25.4 ± 14.0	0.47	0.21
ALT (U/L)	26.4 ± 14.8	22.2 ± 13.1	0.04	26.6 ± 16.4	24.2 ± 11.9	0.20	0.54
γGTP (U/L)	39.6 ± 29.1	40.0 ± 40.2	0.92	32.4 ± 18.9	39.2 ± 38.1	0.03	0.16

A1c, glycated hemoglobin; eGFR, estimated glomerular filtration rate; NT-Pro BNP: N-terminal pro b-type natriuretic peptide

Holter ECG revealed decrease of the Min HR in both groups. However, no intergroup difference was observed in any Holter ECG parameters (Additional file 2).

During the trial period, no significant differences were observed in terms of the dose and oral administration rate of the optimal medical treatment including β-blockers between the two groups (Additional file 3). In the multiple regression analysis of the change in HRV for the empagliflozin group, as items involving SDANN, systolic BP was identified as a significant factor. Single regression analysis revealed that systolic BP was a significant factor for LF and maximum CK was a significant factor for both r-MSSD and HF (Table 4).

**Table 4:** Multiple Regression Analysis of the Change in HRV for the Empagliflozin Group

Secondary evaluation items	Single regression			Multiple regression	
	Regression coefficient	P	Partial regression coefficient	95% confidence interval	P
SDANN					
Systolic blood pressure	0.860	< 0.001	1.235	0.774–1.697	< 0.001
Hematocrit	0.115	0.447			
r-MSSD					
Maximum CK	0.003	0.01			
Hematocrit	0.188	0.212			
HF					
Maximum CK	0.421	0.002			
Hematocrit	0.174	0.248			
LF					
Systolic blood pressure	36.859	0.02			
Hematocrit	0.146	0.334			

SDANN, Standard deviation of all 5 min mean normal RR intervals; r-MSSD, Square root of the mean of the sum of the squares of differences between adjacent RR intervals; HF, high-frequency power; LF, low-frequency power; CK, creatine kinase

#### Safety

During the trial period, two patients in the placebo group met the exclusion criteria and were withdrawn from the trial (including poor blood glucose control and hospitalization for exacerbation of heart failure). No adverse events were observed in the empagliflozin group. There were no deaths, MI recurrence, or stroke onset in both the groups (Additional file 4).

## Discussion

In the EMBODY trial, intragroup comparison revealed a significant improvement in parameters reflecting both sympathetic and parasympathetic nerve activities in the empagliflozin group, whereas intergroup comparison showed no significant difference between the empagliflozin and placebo groups. To be more precise, SDANN, r-MSSD, and HF, all of which reflect cardiac parasympathetic nerve activity, showed significant increases (improvement) in the empagliflozin group, but not in the placebo group. LF/HF ratio, which reflects cardiac sympathetic nerve activity, significantly decreased (improved) only in the empagliflozin group. HRT, which also reflects cardiac sympathetic and parasympathetic nerve activity, significantly improved in the empagliflozin group, but not in the placebo group. Whereas intergroup comparison showed no significant difference between the empagliflozin and placebo groups in the HRT.

As reflected in the results of SDNN and MIBG myocardial scintigraphy, both cardiac sympathetic and parasympathetic nerve activities improved in both the groups in the natural course of AMI. The EMBODY trial demonstrated that these effects might be enhanced by empagliflozin, which is an SGLT2 inhibitor. Conversely, we found no significant difference in LP as an indicator of depolarization or TWA as an indicator of repolarization abnormality. Although experimental data and clinical case reports have suggested that SGLT2 inhibitors improved cardiac sympathetic hyperactivity [16, 17], the present study, to the best of our knowledge, is the first trial to provide randomized clinical data demonstrating that empagliflozin improved both the cardiac sympathetic and parasympathetic activities in humans. The evidence connecting the autonomic nervous system to life-threatening arrhythmias and cardiovascular mortality is well established [18–20]. HRV is a physiological phenomenon characterized by beat-to-beat variations in cardiac cycle length, which is influenced by both sympathetic and parasympathetic autonomic tones. Abnormal HRV, both increased sympathetic and decreased parasympathetic activities, is presently considered a strong predictor for mortality and lethal ventricular arrhythmias in post-MI patients [21]. Several studies have reported that depressed HRV is associated with adverse outcomes in survivors of AMI [22]. Unlike depressed left ventricular ejection fraction, abnormal HRV predicts arrhythmic rather than nonarrhythmic mortality [23].

To date, HRT had been primarily examined in post-MI patients, and it is suggested that abnormal HRT is associated with increased mortality after MI [24]. Therefore, HRV and HRT are surrogate markers for fatal arrhythmias and sudden death, which were improved by empagliflozin in the present study.

Three major mechanisms were considered, which may underlie the improvement in HRV and HRT reflecting cardiac sympathetic and parasympathetic nerve activities with empagliflozin, an SGLT2 inhibitor.

The first mechanism is a hemodynamic effect. It is proposed that improved cardiac sympathetic nerve activity with SGLT2 inhibitors is due to reduced circulating intravascular volume through their osmotic diuresis and natriuresis [25]. This is reflected by an increase in hematocrit, which has also been found to be a key determinant of HF outcomes according to a recent exploratory analysis of the EMPA-REG OUTCOME trial [26]. It is hypothesized that the sustained reduction in intravascular volume and BP lead to a reduction in cardiac preload and afterload, respectively, thereby alleviating cardiac workload and improving LV function [25]. Such hemodynamic changes in intravascular volume and BP are observed without an increase in heart rate, suggesting that SGLT2 inhibitors reduce reflex sympathetic nerve hyperactivity or influence other neurohormonal pathways affecting the heart [27, 28]. In particular, SGLT2 inhibitors reduce BP after 24 h; therefore, we believe that reduced BP led to the reduced cardiac sympathetic nerve activity [29, 30]. In the present study, BP was decreased, but heart rate did not change, which may result in the reduced cardiac sympathetic nerve activity.

The second mechanism is a myocardial energy supply effect. SGLT2 inhibitors have been reported to increase circulating levels of ketone bodies [31]. Ketones are freely taken up by myocardial cells, and may be a more efficient source of adenosine triphosphate for the failing heart compared with fatty acids [32]. Furthermore, it has been found that the utilization rate of ketone bodies is reduced during the course of myocardial ischemia [33]. Studies have suggested that increasing the use of ketone bodies, fatty acid, and branched-chain amino acids inhibited adenosine triphosphate (ATP) reduction, increasing ATP content in the myocardium in the empagliflozin group [34]. In the present study, the serum ketone bodies were also increased in the empagliflozin group, which may have resulted in the decreased myocardial oxygen consumption and decreased cardiac sympathetic nerve activity in patients with AMI being administered empagliflozin. In addition, SGLT2 inhibitors have been reported to increase in erythropoietin, which in itself may have cardioprotective effects, and to increase in hemoglobin, which may result in enhanced oxygen delivery to the myocardium [35, 36].

Furthermore, an emerging hypothesis suggests that SGLT2 inhibitors directly inhibit the myocardial sodium-hydrogen (Na^+^/H^+^) exchanger, thereby leading to increased mitochondrial calcium levels, improved mitochondrial function, reduced oxidative stress, and potentially reduced arrhythmias [37].

The third mechanism is a hepatic vagus nerve-mediated effect. The vagus nerve in the liver controls the activation of neurons in the rostral raphe pallidus (rRPa), which increases the cardiac sympathetic nerve activity, and heart rate [38]. The administration of SGLT2 inhibitors can reduce cardiac sympathetic nerve activity by reducing rRPa activity and in turn control the heart rate [39].

Furthermore, SGLT2 inhibitors reportedly reduced cardiac functioning and size of the infarction in a basic experimental model of AMI [40]. It has been reported that the activation of signal transducer and activator of transcription 3 (STAT3) is the underlying mechanism, which consequently exhibits antioxidative and anti-inflammatory activities [40]. This mechanism may be associated with the reduced cardiac sympathetic nerve activity.

Glycemic variability is associated with cardiovascular autonomic neuropathy [41, 42], and hyperglycemia results in ventricular tachycardia in patients hospitalized with AMI [43]. Moreover, the presence of T2DM without AMI is independently associated with an increase of all-cause mortality in patients presenting with ventricular tachyarrhythmias on admission [44]. In the EMBODY trial, glycemic control levels were even in the two groups. We consider that the shift in blood glucose had very little impact on the improvement of sympathetic and parasympathetic activities with empagliflozin in the present study. In another study, dapagliflozin reduced mean arterial pressure without changing heart rate or sympathetic activity, whereas gliclazide did not have any effect [45]. Previous basic studies have reported that dapagliflozin reduced the sympathetic marker and suppresses prolonged ventricular repolarization [46, 47]. Indeed, SGLT2 inhibitors have been shown to affect several other common modifiable risk factors and comorbidities associated with cardiovascular diseases, such as BW, renal function, uric acid level, and plasma lipid level [1–3, 48, 49]. Taken together, the results of the present study were comparable with those of previous studies [1–3]. The early initiation of SGLT-2 inhibitor therapy should be considered in patients with T2D and established CVD [50].

In terms of safety, SGLT2 inhibitors are not devoid of undesirable effects related to marked glycosuria, such as genital infections, volume depletion, diabetic ketoacidosis (rare), and Fournier’s gangrene (extremely rare) [1–3]. In the present study, no such side effects were observed during the trial period, and no subjects discontinued the trial in the empagliflozin group.

### Trial limitations

First, the trial period was 24 weeks. In the EMPAREG OUTCOME trial, reductions in CV death were observed during an early follow-up period (0–24 weeks), which may be due to decreased SCD with empagliflozin during this period. Thus, we hypothesized that the assessment period of 24 weeks adequately demonstrates the effects of empagliflozin on cardiac nerve activity as a surrogate of lethal ventricular tachyarrhythmias. Second, this trial was conducted only in the Japanese population. However, Japanese patients with coronary artery disease generally receive adequate conventional therapy, including statins. Therefore, it was possible to determine the exact therapeutic effects of empagliflozin against residual risk. β-blockers were not restricted during the trial period. Third, the baseline values of HRV and HRT are somewhat different between the two groups; however, they were not statistically significant. Moreover, the LF/HF ratio has improved with empagliflozin administration in the group with higher HRV, and there is no doubt that this reflects the improvement in cardiac sympathetic nerve activity. Finally, this study was conducted in a limited group with an earlier introduction of SGLT2 inhibitor 2 weeks after AMI onset and a follow up for only 24 weeks. Further studies with an introduction of SGLT2 inhibitor at different timing and a longer follow up period are needed to evaluate whether SGLT2 inhibitors affect cardiac sympathetic nerve activity or not in patients with AMI and T2DM.

## Conclusions

The empagliflozin group might be exhibited a significant improvement in both cardiac sympathetic and parasympathetic nerve activities in the HRV and HRT. Further studies are needed on the use of SGL2 inhibitors for the purpose of reducing cardiac sympathetic nerve activity in patients with AMI and T2DM.

## Supplementary information

**Additional file 1:** Changes in parameters in the 123i-meta-iodobenzylguanide from baseline to 24 weeks.

**Additional file 2:** Changes in the Holter ECG parameters from baseline to 24 weeks.

**Additional file 3:** Dose and oral administration rate of the β-blockers.

**Additional file 4:** Safety parameters in the two groups.

## Data Availability

The datasets are available from the corresponding author on reasonable request.

## Abbreviations

AMI: Acute Myocardial infarction
ATP: Adenosine Triphosphate
BP: Blood Pressure
BW: Body Weight
CK: Creatine Kinase
CV: Cardiovascular
ECG: Electrocardiography
eGFR: Estimated Glomerular Filtration Rate
HF: High-Frequency
HRT: Heart Rate Turbulence
HRV: Heart Rate Variability
LF: Low-Frequency
LP: Late Potentials
MIBG: 123I-*meta*-iodobenzylguanide
MMRM: Mixed-effects Model Repeated Measures
SCD: Sudden Cardiac Death
SGLT2: Sodium–Glucose cotransporter 2
T2DM: Type 2 Diabetes Mellitus
TO: Turbulence Onset
TS: Turbulence Slope
TWA: T-wave Alternans
