# Supplementary material for: Effects of empagliflozin versus placebo on cardiac sympathetic activity in acute myocardial infarction patients with type 2 diabetes mellitus: the EMBODY trial
Source: Cardiovasc Diabetol. 2020 Sep 25;19:148. doi: 10.1186/s12933-020-01127-z (PMC7519555; doi:10.1186/s12933-020-01127-z)
Supplement: Supplementary file 1 — Additional file 1: Changes in parameters in the 123i-meta-iodobenzylguanide from baseline to 24 weeks. [file 12933_2020_1127_MOESM1_ESM.docx]

**Additional File 1.** Changes in parameters in the 123i-*meta*-iodobenzylguanide from baseline to 24 weeks

| **Parameter** |  | **Empagliflozin (n = 24)** | |  | **Placebo (n = 22)** | |  | **Intergroup** |
| --- | --- | --- | --- | --- | --- | --- | --- | --- |
|  |  | **Baseline** | **24 weeks** | **P** | **Baseline** | **24 weeks** | **P** | **P** |
| Heart-to-mediastinum ratio (H/M ratio) early phase | | 2.63 ± 0.51 | 2.70 ± 0.41 | 0.46 | 2.35 ± 0.52 | 2.46 ± 0.58 | 0.06 | 0.65 |
| H/M ratio delayed phase | | 2.63 ± 0.61 | 2.82 ± 0.60 | 0.02 | 2.19 ± 0.66 | 2.37 ± 0.74 | 0.009 | 0.99 |
| Wash out rate (%) | | 28.37 ± 7.48 | 24.68 ± 8.14 | 0.006 | 32.52 ± 9.27 | 28.3 ± 10.12 | <0.001 | 0.63 |
|  |  |  |  |  |  |  |  |  |
