# Supplementary material for: Effects of empagliflozin versus placebo on cardiac sympathetic activity in acute myocardial infarction patients with type 2 diabetes mellitus: the EMBODY trial
Source: Cardiovasc Diabetol. 2020 Sep 25;19:148. doi: 10.1186/s12933-020-01127-z (PMC7519555; doi:10.1186/s12933-020-01127-z)
Supplement: Supplementary file 2 — Additional file 2: Changes in the Holter ECG parameters from baseline to 24 weeks. [file 12933_2020_1127_MOESM2_ESM.docx]

**Additional File 2.** Changes in the Holter ECG parameters from baseline to 24 weeks

| **Item** | **Empagliflozin (n = 46)** | |  | **Placebo (n = 50)** | |  | **Intergroup** |
| --- | --- | --- | --- | --- | --- | --- | --- |
|  | **Baseline** | **24 weeks** | **P** | **Baseline** | **24 weeks** | **P** | **P** |
| THB, beats/day (SD) | 100343.9 (15634.1) | 99432.1 (14695.5) | 0.63 | 102225.5 (15858.0) | 99186.6 (11041.0) | 0.15 | 0.45 |
| Max HR, bpm (SD) | 103.9 (17.0) | 103.8 (15.8) | 0.97 | 108.0 (21.2) | 106.9 (17.8) | 0.68 | 0.76 |
| Mean HR, bpm (SD) | 70.3 (11.0) | 69.6 (10.7) | 0.60 | 71.5 (11.4) | 69.6 (8.0) | 0.20 | 0.53 |
| Min HR, bpm (SD) | 55.7 (8.0) | 53.7 (7.6) | 0.02 | 56.2 (8.7) | 53.7 (6.1) | 0.02 | 0.75 |
| PVC, beats/day (SD) | 431.1 (1464.9) | 388.8 (1003.9) | 0.81 | 119.0 (279.3) | 314.1 (767.3) | 0.08 | 0.24 |

THB; total heart beats, PVC; premature ventricular contraction
