# Supplementary material for: Effects of empagliflozin versus placebo on cardiac sympathetic activity in acute myocardial infarction patients with type 2 diabetes mellitus: the EMBODY trial
Source: Cardiovasc Diabetol. 2020 Sep 25;19:148. doi: 10.1186/s12933-020-01127-z (PMC7519555; doi:10.1186/s12933-020-01127-z)
Supplement: Supplementary file 3 — Additional file 3: Dose and oral administration rate of the β-blockers. [file 12933_2020_1127_MOESM3_ESM.docx]

**Additional File 3.** Dose and oral administration rate of the β-blockers

| **β-blocker** | | **Empagliflozin** | | | | **Placebo** | | | | |  |
| --- | --- | --- | --- | --- | --- | --- | --- | --- | --- | --- | --- |
|  | **Time point** | **Number of patients (n)** | **Mean (mg)** | | **Standard deviation** | **Number of patients (n)** | **Mean (mg)** | | **Standard deviation** | | **P** |
| Cavedilol | |  |  | |  |  |  | |  | |  |
|  | Baseline | 23 | 5.3 | | 3.2 | 24 | 5.4 | | 4.2 | | 0.93 |
|  | 4 Weeks | 23 | 5.3 | | 3.2 | 24 | 5.4 | | 4.2 | | 0.93 |
|  | 12 Weeks | 23 | 5.5 | | 4.0 | 24 | 5.4 | | 4.2 | | 0.92 |
|  | 24 Weeks at discontinuation | 23 | 5.5 | | 3.9 | 25 | 5.4 | | 4.1 | | 0.90 |
| Bisoprolol | |  |  | |  |  |  | |  | |  |
|  | Baseline | 18 | 2.2 | | 1.2 | 14 | 1.6 | | 0.8 | | 0.44 |
|  | 4 weeks | 18 | 2.2 | | 1.2 | 14 | 1.6 | | 0.8 | | 0.44 |
|  | 12 weeks | 18 | 2.2 | | 1.2 | 13 | 1.6 | | 0.8 | | 0.44 |
|  | 24 weeks at discontinuation | 18 | | 2.2 | 1.2 | 13 | | 1.6 | | 0.8 | 0.44 |
