# Supplementary material for: Effects of empagliflozin versus placebo on cardiac sympathetic activity in acute myocardial infarction patients with type 2 diabetes mellitus: the EMBODY trial
Source: Cardiovasc Diabetol. 2020 Sep 25;19:148. doi: 10.1186/s12933-020-01127-z (PMC7519555; doi:10.1186/s12933-020-01127-z)
Supplement: Supplementary file 4 — Additional file 4: Safety parameters in the two groups. [file 12933_2020_1127_MOESM4_ESM.docx]

**Additional File 4.** Safety parameters in the two groups

|  |  | **Empagliflozin** | | **Placebo** | |
| --- | --- | --- | --- | --- | --- |
|  |  | **n = 46** | | **n = 50** | |
|  |  | **Number of cases, n** | **Number of patients, n (%)** | **Number of cases, n** | **Number of patients, n (%)** |
|  | Overall | 0 | 0 | 5 | 5 (10.0) |
|  | Dizziness | 0 | 0 | 1 | 1 (2.0) |
|  | Heart failure | 0 | 0 | 1 | 1 (2.0) |
|  | Abdominal pain | 0 | 0 | 1 | 1 (2.0) |
|  | Hepatic impairment | 0 | 0 | 1 | 1 (2.0) |
|  | Rash | 0 | 0 | 1 | 1 (2.0) |
